# Simulated patient’s feedback to improve communication skills of clerkship students

**DOI:** 10.1186/s12909-019-1914-2

**Published:** 2020-01-15

**Authors:** Ayesha Aleem Qureshi, Tabassum Zehra

**Affiliations:** 1 Department of Health Professions Education, Al-Nafees Medical College & Hospital, Isra University, Islamabad Campus, Islamabad, Pakistan; 2 Department of Educational Development, Aga Khan Medical College, Aga Khan University, Karachi, Pakistan

## Abstract

**Background:**

The changing trends of the society and revisions to medical education have changed the way medical students are trained to adroitly care for patients hence, patient centered care has become need of today’s society and communication skills are imperative in developing patient physician relationship. Increasingly, simulations are being used to aid medical students to incorporate theoretical knowledge into practice. There are innumerable studies regarding communication skills in terms of reliability, validity and feasibility but no such study has been documented using simulated patient’s feedback in improving communication skills in Pakistan. The aim of this study is to explore whether simulated patients’ feedback improves the communication skills of undergraduate medical students.

**Methods:**

During a randomized control trail a group of eighty students in the final year clerkship at Al-Nafees Medical College have participated in pre-post Objective Structured Clinical Exam (OSCE) on communication skills. The students were selected through convenience sampling technique. Four Objective Structured Clinical Exam (OSCE) stations based on different scenarios of communication skills were developed. Each station of fifteen minutes duration was assessed by both simulated patients and faculty using a validated tool LCSAS (Liverpool Communication Skills Assessment Scale). The difference between the pre and post-tests of two groups was explored by applying independent t-test. Cronbach’s alpha was used to check the reliability of scores and effect size was calculated.

**Results:**

Results of this study have showed that there is significant improvement in communication skills after receiving feedback from simulated patients (*p* value ≤0.05) was observed. An overall Cronbach α = 0.83 on LCSAS reveal a high internal consistency and there was adequate demonstration of effect size(r = 0.8).

**Conclusion:**

The results on the scores of the students on the Liverpool Communication Skills Assessment Scale confirm that simulated patient’s feedback is essential to enhance the communication skills of the medical students. This study offers significant evidence towards successful conduction of a formal communication skills development initiative at Al-Nafees Medical College using simulated patient feedback during teaching and assessments.

## Background

Patient centered care is gaining prime importance globally [1]; it is defined as giving priority and respect to patient’s desires, requests and preferences, so patient can choose the treatment plan that best fit their personal needs [2]. This egalitarian approach is unlike the doctor-centered or paternalistic approach, which encourages patient to play role of a partner [3] and makes them more accountable for their own health [4].

Communication skills have a key role in patient centered care as it influences compliance to treatment plan and patient satisfaction [5]. The modern clinical conversation has become increasingly complex, as doctors explain to the patients about drug regimes, its side effects, motivate them on their lifestyle, coordinate with other specialists [1] and also negotiate with health insurance companies [6]. Expertise in Communication have become an imperative demand for doctors worldwide, according to the Accreditation Council for Graduate Medical Education [7], the American Board of Medical Specialties [8], the Association of American Medical Colleges [9], the General Medical Council [10], and the World Federation for Medical Education [11], communication and interpersonal skills are among the fundamental competencies to be taught in medical and residency programs. The World Health Organization (WHO), in its proposed ‘Five Star Doctor’ emphasizes the need of a good communicator to be one of the essential competencies of graduating student [12] and now Pakistan Medical and Dental Council [13] have also stressed on teaching communication skills in curriculum for undergraduate MBBS program.

There is a dire need to enhance the communication skills amongst Pakistani medical students to meet the global criteria of patient centered doctors [14]. Until now, assessment of student’s communication skills is in a preliminary stage in most of the medical schools in Pakistan [15] .It is a difficult task due to two main reasons; an increase in the number of medical students and additional responsibilities of the faculty such as educational work, research and health services. Many physicians have only a limited repertoire of effective communication skills or are too busy to concentrate on communication skills shown by each student. Due to lack of proper training system, students are left to learn communication skills by themselves this leads to poor communication skills and dissatisfaction amongst patients [16].

Various reasons have been proposed suggesting that it is imperative to assess communication skills specifically. It has been observed that students only learn through assessment and pay no heed to goals which are not assessed. Students can identify their own learning needs by receiving feedback about their communication skills through assessments. By rating the students the faculty also gets trained, it enhances their own performance and can improve in teaching communication skills. Lastly, evaluation and modification of curriculum becomes easier [17].

### Simulated patients

Simulated patients and standardized patients are most frequently used terms: both abbreviated as (SP) were introduced by Barrows in sixties [18]. A standardized patient has been described as an umbrella term for both a real patient and a simulated patient. A real patient is one who is trained to present his or her own illness in a standardized way. Simulated patient is a person who has gone through rigorous training, to portray signs and symptoms of real patients.

Nowadays, simulated patients (SPs) are used globally to rate history taking, physical examination and communication skills [19]. It is widely accepted that SPs are practical, economical, dependable and legitimate means to assess clinical aptitude of students [20, 21] moreover, they can be trained to provide constructive feedback to students [18] during teaching sessions and assessments. The use of simulated patients’ pedagogy in healthcare field, have reported changes in increase in knowledge, attitude and learner’s satisfaction [22] and have shown equivalent or even better communication and examination skills, compared to the students only trained by faculty [23–27].

### Role of feedback by simulated patients

The main advantage of using simulated patients is the feedback provided to the students from a patient’s perspective [19, 28]. Feedback can be defined as “explicit guidance about the assessment between trainees’ observed performance and a standard, given with an intention to improve student’s performance” [29, 30]. Immediate feedback written or oral provided by SPs after a student encounter is extensively used in several medical schools [31]. Feedback provided by the simulated patients is appreciated just as or more positively than the feedback provided by doctors [32, 33]. A prodigious advantage to medical students is that they can remarkably enhance their communication skills in a non-threatening environment before experiencing the complexity of “real” patients. The student can rectify mistakes through patient feedback which will boost their self-confidence.

In local context of Pakistan, literature reports a study that compared the effectiveness of simulated patients with real patients through mini-CEX to assess communication and clinical skills from student’s perspective [34]. Another study conducted at Fatima Memorial Hospital (FMH) Medical and Dental College, where counseling skills were assessed of fourth year medical students using pre-recorded videos and role play showed an improvement in the communication skills of the medical students [14]. At Aga Khan University researchers compared examiner assessment with student self-assessment on communication skills using same rating scale on three OSCE stations [35]. All these studies stressed that in order to strengthen the clinical curriculum it is mandatory to include feedback. There is a dearth of literature on using simulated patient’s feedback in improving communication through teaching and assessment at undergraduate medical colleges in Pakistan [36].

Although communication skills are being taught in all medical undergraduate curricula, there is no comprehensive program of assessing communication skills using professionally trained Simulated Patients in Pakistan. The study will lead to a new strategy in teaching and assessing student’s communication skills.

### Research question

Does the simulated patient’s feedback have an effect on OSCE performance of student’s communication skills?

### Hypothesis

Alternate Hypothesis: Simulated Patient’s feedback has a significant difference in the mean OSCE scores on clerkship student’s communication skills performance in the OSCE.

## Methods

A single blinded study was conducted at a private medical college (Al-Nafees Medical College). Communication skills of eighty students of final year clerkship were assessed using an experimental study design. All students of final year clerkship at ANMC were invited to participate in the study however; only eighty students gave their consent and took part in the study.

### Design and duration

In this 6 months experimental study, the communication skills of eighty students of final year clerkship were assessed using an experimental study design. A Randomized Control Trial (RCT) was used in this study one group is randomly allocated to the intervention group and the other group is randomly allocated to the standard group (control group). The difference measured in the study is the outcome.

### Sample size and setting

Eighty (80) students out of hundred (100) students of Final Year MBBS clerkship (Year 5, 2017) studying at Al-Nafees Medical College, Isra University participated in this study.

### Sampling technique

Convenient sampling was done in this study.

### Variables

- Dependent variable: Student scores on OSCE
- Independent variable: Feedback provided by simulated patients

### Randomization and group allocation

Out of the hundred students, eighty students who volunteered for the study were included, the subjects were randomly assigned to either the experimental (Group “A”) or the control (Group “B”) from roll number list. The groups were balanced with respect to number allocation. Control over gender mix was not possible due to systematic bias. At Al-Nafees Medical College, the male and females are segregated into four different study groups two are for male students while other two are for female students as per institutional policy. Therefore, although each group had both boys and girls but the numbers were slightly different (in total 39 male and 41 female students participated in the study). All students who gave their consent were called group wise and were randomly assigned to experimental or control group. The students in this study were of same class, institute and age groups with no prior training sessions on communication skills. Furthermore, the students were blinded to the SP’s and the faculty assessing each Objective Structured Clinical Exam station (OSCE). The students were randomly assigned from the roll number list to groups at the spot so they were ignorant whether they were part of experimental or control group. Students had their first interaction with the SPs during OSCEs.

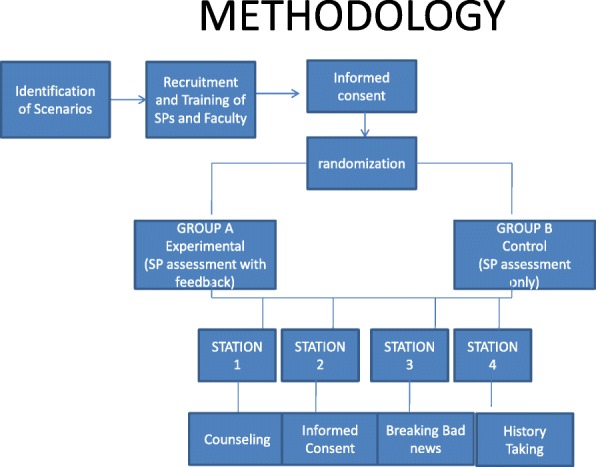



### Inclusion criteria

All students of final year clerkship at ANMC were invited to participate in the study. Only those students were included in the study who gave their consent.

### Exclusion criteria

All those students who were not part of final year clerkship were excluded from the study.

### Training

Simulated patients should be “standardized” in their performances so students can get the same test experience [18]. This requires that simulated patients initiate the encounter in concise, well scripted and timely manner. Any symptomatic information would only be provided by SP when asked by the medical student and the SP replies without unnecessary explanation [18, 37, 38]. All these points were given due consideration during the training session.

A week before the first OSCE session, total twelve individuals which included six faculty members who were trained as assessors and other three students and three employees from other departments at Isra University, Islamabad were trained as Simulated Patients. A one-day workshop was developed and delivered to all twelve individuals by three subject experts (Psychiatrist, Clinical Psychologist and a Researcher) having years’ experience in teaching communication skills at ANMC.

Apart from the one-day workshop, individual training sessions (one to two) were given to the SP’s (students and employees) through role-plays on the scenarios of OSCE stations.

The six candidates selected other than the faculty, as simulated patients were studying and working at Isra University. Out of the six SP’s, three males participating in the study were students of final year ‘Vision Sciences’ while three females were working in the department of Physiotherapy. Participants’ were in the age group between mid 20’s and mid 30’s. All the candidates self-volunteered for the study as they had keen interest in communication skills. Since they also interact with patients they were highly motivated to improve their own communication skills. In spite of the wide variations in age, gender and education the simulated patients created a unique atmosphere of laughter, debate, and sarcasm. Out of the six trained, four SP’s were used in the study. Two simulated patients were trained as standby in case of any emergency if anyone was unavailable they can substitute for them.

Similarly six faculty members were also trained as assessors keeping extra two members in case of any emergency. Same SP’s were used for both sessions of pre and post-test OSCEs.

The workshop was designed on the format of “Focusing Feedback on Interpersonal Skills”: A Workshop for Standardized Patients (Facilitator’s Guide by LD Howley). The workshop was planned as a single learning experience, lasting three hours. The first segment of the workshop started with the purpose of feedback, in general, and specifically what role does feedback play in the success of the SP program? Participants were taught about Constructive feedback which should be both non-evaluative and descriptive and the “DESC” technique (describes your behavior ***(D) When,*** express your feelings ***(E) I felt,*** specify the desired change in behavior ***(S) I’d prefer***, communicate any consequences ***(C) Communicate Consequences)***. DESC technique was originally developed by Bower & Bower [39].

In the next phase simulated patients were educated on the therapeutic Communication Skills. Both verbal and nonverbal portions were comprehensively explained to all participants with demonstrations. It was advised that vocabulary should be appropriate according to the patient’s level of understanding; Patients should be greeted by name; Physician should initiate self-introduction; Explanations should be organized and clear; Physician should use restatement, reflection, and clarification techniques to convey active listening and ensure accuracy of interpretation. Voice tone should be caring and empathetic. Voice volume should be appropriate (not too loud or too soft).

A lot of stress was given to non-verbal communication. Many researchers have estimated that 95% or more of the messages we convey to others are communicated non-verbally. Eye contact should be appropriate, facial expressions should be congruent with content of speech, appropriate gesturing not excessive should be practiced, use for emphasis and demonstration were explained, proximity which is keeping comfortable distance from the patient was explained, ideal posturing and positioning, appropriate use of touch was discussed. Focus of attention should always be on the patient was highlighted. Simulated patients were trained on difference between open and close ended questions, how to summarize and empathize with patients. Lastly, it was stressed to ensure patient understanding. The workshop comprised of interactive session on all aspects of communication skills where both the participants and facilitators discussed the topics with each other. Liverpool Communication Assessment Scale was explained and discussed followed by role-plays. The role-plays were based on the scenarios of the four OSCE stations on communication skills. The verbal feedback given in the role-plays was through DESC technique.

After the training session in which simulated patients learned their character scripts, simulated patients along with the assessors return to the demo OSCE station for inter-rater reliability training. The faculty and the other simulated patients watched the performance. At the conclusion of each role-played performance, the experts, faculty and simulated patients each completed their assessment on Liverpool Communication Assessment Scale (LCSAS).

In this study simulated patient’s feedback is the intervention being used hence; a lot of emphasis was dedicated to this area.

### Data collection tool

The data was collected through Liverpool Communication skills Assessment Scale (LCSAS) which has been developed in UK. The scale has an open access and permission to use. The Liverpool Communication Skills Assessment Scale has earlier been used in a study on Objective Structured Video Exam for assessment of communication skills at the University of Liverpool [40] and literature supports that LCSAS is a reliable tool with an acceptable validity [41]..

Kalamazoo standards on communication are based on the Bayer-Fetzer Conference on Physician-Patient Communication in Medical Education report [42] which identified seven key elements of communication in clinical encounters: build relationship, open discussion, gather information, understand patient’s perspective, share information, reach agreement, and provide closure [42].

The Liverpool communication skills assessment scale is based on five fundamental core competencies namely:

- Introduction: Having items on greetings and check patients’ identity and introduction of self and role (two items).
- General: Having items on audibility and enunciation, eye contact and nonverbal facilitation (three items).
- Respect and empathy: Having items on respect the patient and reflects empathy to patient’s feelings (two items).
- Questioning: Having items on appropriate open and closed questions, clarification questions and summarizing and sensitivity of questions (three items)
- Giving information: Use of clear language, ensure understanding and close appropriately (two items).

The LCSAS is a 12-item measure designed to assess several aspects of communication skills in medical students. It is on a four–point Likert scale where 0 = unacceptable, 1 = poor, 2 = acceptable and 3 = good. The measure was designed to be completed by an examiner during Objective Structured Clinical Examinations (OCSEs) and assesses five domains of communication, including introduction, nonverbal behavior, respect and empathy, questioning, and giving information. The purpose of the tool is twofold:

- To assess communication skills in a standardized fashion.
- To provide personalized feedback on observed communication skills.

Liverpool communication skills assessment scale is appropriate for this study as it is a validated tool. It is easy to be used by simulated patients as it is operationally defined and is convenient considering the limited time available for OSCEs.

### Data collection method

Standardized patients rated the student’s ability to keep a patient-centered approach across four diverse communication tasks by understanding the patient’s perspective, being involved in exploring the patient’s feelings, ideas, concerns, and experiences regarding the impact of the illness, and giving consideration to patients expectation from a physician. These assigned tasks were based on a review of communication skills literature that includes:-

- Giving bad news
- Counseling
- Conducting a focused history and
- Informed consent

SP’s and the faculty used the same validated communication skill tool LCSAS individually to assess the student’s communication skills. Each of the 4 stations started with a 5-min encounter with the simulated patient. During a post-encounter interval, the Simulated Patient completed a 12-item (4-point) validated (LCSAS) rating scale based on Kalamazoo standards. At the end of the post-encounter, the SP debriefs the encounter and give five minutes of verbal feedback to the student. Only group “A” received verbal feedback from the trained SP while remainder of the class received feedback at the end of the study. The scored LCSAS were collected back from the SP’s and the faculty. The post test was conducted after 2 weeks. During posttest each of the 4 stations started with a 5-min encounter with the simulated patient. During a post-encounter interval, the Simulated Patient completed a 12-item (4-point) validated (LCSAS) rating scale. Each station was rated by a trained SP and a faculty member using the same scale.

Data triangulation was ensured through two observations at different times (pre and post-test) and through two raters (faculty and SP). For data triangulation the data is collected through multiple sources to assure validity of the research. In this study data was collected by two raters who had separately made their judgments about the student’s communication skills at two different times (pretest OSCE and posttest OSCE). There was no threat to internal validity since there was no change in study design and there were no drop outs.

### Data analysis

The quantitative data was collected from the SP’s assessment of student’s communication skills during OSCE. It was analyzed using Statistical Package for Social Sciences (SPSS) version 20. The data was interpreted using independent *t-*test and comparing the means. Cronbach’s alpha was useful to check the reliability of scores and effect size was analyzed to measure the difference between two groups.

### Ethical considerations

Ethical approval of this study was taken from AKU Ethical Review Committee and the IRB at Al-Nafees Medical College.

## Results

In the study pre and post-test OSCE was conducted for all four stations on communication skills. All participants were divided into two groups through randomization, in this way each group contain equal number of participants.

The reliability (Cronbach’s α) of the Liverpool Communication Assessment Scale (LCSAS) was α = 0.83 overall on four stations. Out of the eighty students thirty nine (48.8%) were male and forty one (51.3%) were female. The participants in study were between an age group (22–24 years).

Table 1 shows that the lowest mean was on item nine ‘clarifying and summarizing’ (mean = 1.00, SD 0.827). The highest mean was on item three ‘general audibility and enunciation’ (mean = 2.52, SD 0.576). Item numbers two ‘introduction of self and role’, item seven ‘empathy’, item eight ‘appropriate open and closed questions’, item nine ‘clarifying and summarizing’, item ten ‘sensitivity of questions’ and item twelve ‘ensures understanding and closes appropriately’ had the mean score < 2 (mean = .100 to 1.97, SD = 0.705 to 0.827. Whereas, item one’ greeting and checks patient identity’, item three ‘general audibility and enunciation’, item four’ eye contact’, item five ‘nonverbal facilitation’, item six ‘respect patient’ and item eleven ‘uses clear language’ had a mean > 2 (mean = 2.01–2.52, SD = 0.303 to0.885).

**Table 1:** Descriptive statistics pre-test OSCE

		N	Mean Min = 0 Max = 3	Std. Deviation
1.	Greeting and checks pt. identity	80	2.08	0.88
2.	Introduction of self and role	80	1.36	0.70
3.	General audibility and enunciation	80	2.52	0.57
4.	Eye contact	80	2.26	0.47
5.	Nonverbal facilitation	80	2.21	0.52
6.	Respect patient	80	2.01	0.30
7.	Empathy	80	1.60	0.71
8.	Appropriate open and closed questions	80	1.97	0.64
9.	Clarifying and summarizing	80	1.00	0.82
10.	Sensitivity of questions	80	1.95	0.66
11.	Uses clear language	80	2.06	0.67
12.	Ensures understanding and closes appropriately	80	1.08	0.72

Table 2 shows that the lowest mean was on item eleven ‘uses clear language’ (mean = 2.25, SD 0.803). The highest mean was on item four ‘eye contact’ (mean = 2.52, SD 0.644). Item numbers two ‘introduction of self and role’, item seven ‘empathy’, item eight ‘appropriate open and closed questions’, item nine ‘clarifying and summarizing’, item ten ‘sensitivity of questions’ and item twelve ‘ensures understanding and closes appropriately’ item one’ greeting and checks patient identity’, item three ‘general audibility and enunciation’, item five ‘nonverbal facilitation’ and item six ‘respect patient’ had a mean > 2 (mean = 2.30–2.95, SD = 0.601 to 3.904).

**Table 2:** Descriptive Statistics Post Test OSCE

		N	Mean Min = 0 Max = 3	Std. Deviation
1) ++	Greeting and checks pt. identity	80	2.36	0.60
2)	Introduction of self and role	80	2.36	0.80
3)	General audibility and enunciation	80	2.65	0.67
4)	Eye contact	80	2.97	2.62
5)	Nonverbal facilitation	80	2.52	0.64
6)	Respect patient	80	2.48	0.53
7)	Empathy	80	2.35	0.86
8)	Appropriate open and closed questions	80	2.95	3.90
9)	Clarifying and summarizing	80	2.37	0.76
10)	Sensitivity of questions	80	2.49	0.59
11)	Uses clear language	80	2.25	0.80
12)	Ensures understanding and closes appropriately	80	2.30	0.96

### Comparing means

An independent*-*test was conducted to compare student scores on each OSCE station using LCSAS with and without feedback (Table 3).

**Table 3:** Independent sample *t* test: comparison of means of Group A and Group B on Station 1

Stations	Group A Mean	Group B Mean	Mean Difference	Significance
Station 1 counseling	28.91	23.79	5.125	< 0.00
Station 2 Informed consent	26.18	20.78	5.400	**< 0.001**
Station 3 Breaking Bad News	22.59	23.01	−.425	0.453
Station 4 History taking	28.56	23.84	4.725	< 0.001

There was a significant difference for scores of group A and B. As the *p*- value is less than 0.05. The scores of (Group B) mean = 23.79 and with feedback (Group A) mean = 28.91.

Independent sample *t* test: comparison of means of Group A and Group B on Station Two: Informed Consent there was a significant difference for scores on without feedback (Group B) mean = 20.78 and with feedback (Group A) mean = 26.18. The *p*-value of < 0.001 suggests significance of results. Independent sample *t* test: comparison of means of Group A and Group B on Station Three: Breaking Bad News. There was no significant difference for scores on without feedback (Group B) mean = 23.01and with feedback (Group A) mean = 22.59.The results were not significant with two tailed *p* value = 0.453.Independent sample *t* test: comparison of means of Group A and Group B on Station Four: History Taking a significant difference was observed for scores on without feedback (Group B) mean = 23.84 and with feedback (Group A) mean = 28.56.The results were significant with a two tailed *p*-value of < 0.001.

The effect size was calculated using Cohen–d to indicate the standardized difference between two means. The effect size for this study was 0.8 which indicates large effect.

## Discussion

The results on the scores of the students on the Liverpool Communication Skills Assessment Scale (pre and post intervention) confirm that feedback is essential to enhance the communication skills of the medical students, rejecting the null hypotheses which was that Simulated Patient’s feedback has no significant difference in the mean OSCE scores on clerkship student’s communication skills performance in the OSCE.

An overall α = 0.83 of student responses shows that the items on LCSAS have a high internal consistency and this also confirms that LCSAS is a reliable tool in the local context. The tool had a high reliability as compared to the original study with just acceptable reliability (Cronbach’s α = 0.7). This study has highlighted the effectiveness of simulated patient’s feedback in improving the communication skills of undergraduate medical students. Numerous studies have indicated that physician’s communication skills have an immense effect on the nature of physician patient encounter [43–46] and the techniques and style of a physician determines patient satisfaction. Similarly, using simulated patients feedback is considered as an innovative teaching learning strategy [47].

The scores on LCSAS scale used in pre-test revealed lowest scores on item nine ‘clarifying and summarizing’. This may be due to the fact that since currently no training is being provided to the students on communication skills they have performed poorly on this item. On the other hand the highest mean was on item three ‘general audibility and enunciation’. This was one of the simplest item on the rating scale and during pre-test students were able to perform well on this item.

In comparison to the pre-test the post-test by LCSAS showed that the lowest mean was on item ‘uses clear language’. One possible reason may be the habit of using jargons by the medical students which makes understanding difficult for the patients including the simulated patients. The highest mean was on item four ‘eye contact’. After an initial experience during the pre-test OSCE with simulated patients along with feedback overall the students were more confident and were able to score well on this item. The overall results of post-test were much higher than the pre-test OSCE scores which confirm that feedback provided to the students enhanced their communication skills. A similar study reports that achieving competence in undergraduate medical education requires opportunities provided to the students to practice their skills. This may be either on simulation or on real patients. Therefore, many undergraduate and postgraduate curricula around the world are now providing simulated patient encounters [22] .

Empathy is said to be an essential component of medical training and education. However, empathy is reported to decrease as the years of training progress [48]. The station on breaking bad news was the most difficult station as perceived by the students in an informal discussion after the post-test. The higher score of the control (Group B) as compared to the interventional group might be due to the fact that few more female participants were in the control Group B and since they were more empathetic than the male students hence Group B has performed better than group A. This is also in line with an earlier study conducted in Bangladesh [48] .

This study confirms with an earlier study conducted to review articles published on efficacy of simulated patients in developing communication skills. In the study sixteen out of twenty two published articles highlighted that programs or training sessions by simulated patients resulted in students improved performance of communication skills with the use of SP with feedback than those without SPs participation [49] .

Similarly, in a study, Bachmann concluded that undergraduate medical students who were given a concise two-hour communication skills training performed better in a primary care communication examination than students who received no training (*p* = 0.02) [50] which is close to the value in this study (*p* = < 0.001).

Literature supports the notion that teaching with a hybrid model through use of both simulated patients and mannequins may result in better communication skills rather than just through small-group tutorials. This also supports the potential of simulated Patients as an effective additional tool in communication skill development [49]. This can be interpreted with the current study where students scored low in the pre-test on item related to empathy. However, they were able to improve in the post test which confirms that feedback coupled with simulated patient encounter or experience is essential in improving communication skills of future doctors.

Another study compared the effect of communication skills through role play scenarios using simulated patients and didactic lectures only. No major difference between the two groups (*p* = 0.238) [49] .

In the present study the SPs were trained based on the clinical scenarios to provide feedback to the students on OSCE stations. The clinical scenarios were close to real life formulated by the subject experts. This is also in line with another study which reports reflective practice (reflection on real life clinical encounters) may be used to challenge clinical scenarios and rater training in providing feedback. This may augment SP program in identifying strategies to teach and assess students on communication skills [51] .

This four station OSCE provided a reliable and valuable way to assess students and teach them communication skills at Al-Nafees Medical College. As the students were assessed in five minutes encounter during the OSCE and an immediate feedback were provided post encounter. This is in line with the literature that valid and reliable measures of teaching and assessment can guide personal and professional development; determine readiness of the student for independent practice. It also deepens the understanding of the communication itself [52]. Literature also reports that communication skills OSCE stations, including difficult scenarios (beyond history taking) may have acceptable reliability. However, OSCE stations with certain set of case scenarios that can be generalized remains indefinable [52].

The main advantage observed was assessment of challenging scenarios in a controlled setting (OSCE), hence the risk to both patient and student was minimized. Lastly, all students encountered the same case ensuring fairness and enabling direct comparison of all students. The “teaching time” immediately after the encounter in which students are very receptive to reflect on their experience was facilitated by feedback from simulated patients.

Literature reports that the students performances should be assessed according to the their level and complexity of cases, their training and skill [52]. A graduating student is expected to be competent to be able to perform independently the essential tasks of communication (history taking, counseling, informed consent, breaking bad news, patient education and their families) [52]. Hence, assessment methods should be appropriate with valid and reliable tools.

This study provided an evidence based model for incorporating SP’s to provide feedback in teaching and assessment modalities for communication skills at ANMCH.

In future OSCEs result on communication skills can provide valuable information on curriculum review at ANMC. Areas of empathy and use of clear and simple language identified as weakest areas of students can be targeted as priority areas of improvement in the curriculum at ANMC.

## Conclusion

This is the first experimental study (RCT) to the best of author’s knowledge to explore the effects of using simulated patients feedback in teaching and assessing undergraduate medical students in Pakistan.

A gap was observed in behavioral outcomes at ANMCH that can affect clinical practices and patient outcomes. This study has explored this gap and indicates an improvement in the behavioral outcomes of the participants.

This study has provided evidence to support simulated patients feedback in improving communication skills although the exact magnitude of the impact of intervention on communication skills cannot be predicted. However, this study offers significant evidence towards successful conduction of a formal communication skills development initiative at ANMC using simulated patient feedback during teaching and assessments.

### Strengths and limitations

This is the first of its kind of experimental (RCT) published research in the local context to the best of the researchers knowledge to enhance communication skills of undergraduate medical students through simulated patient’s feedback.

There were several limitations in this study. The study was conducted on a small number of students and at one site only therefore, the results cannot be generalized. Secondly, there was an unavoidable contact between the two groups although study was conducted during study break, this may have affected the overall results of the study, since there was gap of around 2 weeks between the initial and final OSCE. Thirdly, there was no follow up to find out the long term effects of using SPs. Lastly, one time training is insufficient multiple sessions with the simulated patients should be used.

## Recommendations

- Initiate a SP patient bank in ANMCH.
- Include SP’s in teaching and assessment of communication skills in undergraduate curriculum.
- Include a longitudinal theme of communication skills from Year 1 level in order to enhance communication skills competence in future doctors.
- Evaluate the curriculum of ANMC through incorporating SP feedback.

## Future research area

- Using variety of simulated patients (adolescents, old aged and transgender) to train undergraduate medical students on communication skills.
- Impact of the feedback provided by SP’s in real life practice of future doctors.
- Multicenter study using SP feedback to enhance communication skills in undergraduate medical education.
- A qualitative study should be carried out to explore the views of undergraduate Pakistani medical students about simulated patient’s feedback on improving communication skills.

## Data Availability

Data cannot be shared as per institutional policy.

## Abbreviations

ANMC&H: Al-Nafees Medical College & Hospital
LCSAS: Liverpool Communication Skills Assessment Scale
OSCE: Objective Structured Clinical Exam
